# The General Practitioner and the Consultant

**Published:** 1907-04-27

**Authors:** 


### Editor's Letter-Box.

### THE GENERAL PRACTITIONER AND THE CONSULTANT.

Sir,—Your leading article, following so closely upon the recent correspondence in the *British Medical Journal*, indicates the feeling of suspicion and distrust which exists among many general practitioners as to the honesty of the consultant.

I am well aware that there are many consultants—yes, and men with big names—who see every patient that comes to them; who put these patients into homes and undertake their treatment without so much as inquiring if they have been under the care of a general practitioner. These "consultants" usually meet with a just retribution sooner or later, and I do not think the general practitioner need seriously worry his head about them. In fact, the punishment of any unprofessional consultant is so entirely in the hands of the general practitioner that he (the G.P.) can hardly be said to have a grievance.

But there is the other side of the question. Has the consultant no cause to complain of the treatment he receives from the general practitioner? As a young consultant, I say he has very serious cause. Twice within the last few days have I been consulted by patients—one coming from the country—without the knowledge of his doctor, and the other with this knowledge and acquiescence—and in each case I wrote a long and explicit letter to the doctor in attendance.

In neither instance have I had so much as an acknowledgment. I shall, however, go on writing to doctors, because it is an act of courtesy from one gentleman to another, if nothing more; and not because I can possibly think it to my advantage to run the risk of a snub!

I am glad to say it is not always so. I make a habit of writing personally to all those (perhaps humbler) members of the profession working among the poorer classes who are good enough to send their difficult cases to hospital to see me—often with shrewd and wise letters of explanation—and from them I invariably get most grateful letters of thanks.

I make, therefore, my serious indictment of discourtesy against those practitioners in lucrative practice who are too puffed up with their own importance to be even civil to their younger and more struggling brethren, many of whom could, perhaps, have been taking the bread from the mouths of these prosperous ones had they not preferred to submerge themselves (for a time) in working out their own salvation, and that of others, in the laboratories and hospital wards, in order to reach that fame which will ensure respect from the most inflated practitioner.

I appeal to these gentlemen not to treat the younger consultants with the scant courtesy so little deserved.

I am yours, etc.,

Spes.

